# The 116G > A MSH6 and IVS1-1121C > T PMS2 Genes Polymorphisms Modulate the Risk of the Sporadic Colorectal Cancer Development in Polish Population

**DOI:** 10.1007/s12253-017-0231-5

**Published:** 2017-04-27

**Authors:** Piotr Zelga, Karolina Przybyłowska-Sygut, Marta Zelga, Adam Dziki, Ireneusz Majsterek

**Affiliations:** 1 Department of General and Colorectal Surgery, Medical University of Lodz, Plac Hallera 1, 90-647 Lodz, Poland; 2 Department of Biochemistry, Medical University of Lodz, Lodz, Poland

**Keywords:** Colorectal cancer, SNP, MSH6, PMS2, DNA MMR

## Abstract

Colorectal cancer (CRC) is one of the most common cancers worldwide. DNA mismatch repair (MMR) is an evolutionarily conserved process that corrects mismatches generated during DNA replication. MMR defects were found to be associated with hereditary non-polyposis colorectal cancer (HNPCC) and a subset of sporadic colon cancers. The inheritance of common variations in MMR genes may influences individual susceptibility to the development of colorectal cancer. The purpose of the study was to evaluate the association between gene polymorphisms Glu39Gly (c.116G > A) of MSH6 gene and IVS1-1121C > T of PMS2 gene and sporadic colorectal cancer risk, in a case-control study comprising 200 patients and 200 controls origination from polish population. DNA was isolated from peripheral blood lymphocytes of enrolled patients, and gene polymorphisms were analysed by restriction fragment length polymorphism-polymerase chain reaction (RFLP-PCR) for MSH6 and TaqMan for PMS2. G/A variant of Glu39Gly (c.116G > A) genotype was associated with an increased risk of colorectal cancer (OR 1,65 95%CI:1,01–2,69 *p* = 0.44). Presence of A allele was also significantly higher in patient with CRC (OR 1,57 95% CI: 1,04–2,38 *p* = 0.032). Prevalence of this genotype was also markedly higher in females and patients above 60 years in CRC group (OR 2.25 95%CI: 1.22–4.14 *p* = 0.0098 and OR 2.74 95% CI: 1.27–5.93 *p* = 0.0097 respectively). None of such correlations was observed for genotype variants of IVS1-1121C > T PMS2. In conclusion, our data suggests thatMSH6 Glu39Gly polymorphism is associated with the risk of developing sporadic colorectal cancer in polish population. Linkage to the female gender, onset above 60 years old and further increase of risk when combined with wild-type allele of PMS2 IVS1-1121C > T polymorphism indicates defective mismatch repair system.

## Introduction

Colorectal cancer (CRC) is the third most common cancer in the world, with nearly 1.4 million new cases diagnosed in 2012 [1]. It is predicted that worldwide the number of cases will rise to 1.36 million for men and 1.08 million for women by 2035 [2]. CRC represents heterogeneous groups of malignant tumours evolving through multiple pathways. Actually, three different, but partly overlapping, molecular phenotypes reflecting different forms of DNA instability has been proposed including chromosomal instability (CIN), microsatellite instability (MSI) and CpG island methylator phenotype (CIMP) [3, 4]*.* Almost 85% of all CRC arise through CIN pathway, whereas remaining ones are subject to MSI. MSI results from inefficient function of DNA mismatch repair (MMR) system, comprising of MSH2, MSH6, MSH3, MLH1, PMS2, PCNA and EXO1 proteins [5]. Deficient MMR system resulting in high-MSI accounts for 15% of CRCs with 3% being associated with hereditary non-polyposis colorectal carcinoma (HNPCC), and the 12% with sporadic cancer [6], but low-MSI status is often noted in group of tumours originating from other pathways [3, 7]. This indicates that MSI status may be involved in significantly larger number of CRC cases than those confined only to MSI-H phenotype. The efficiency of MMR system may be modulated not only by germline mutations or hypermethylation of the MLH1 promoter, but also through the genetic variations known as single nucleotide polymorphisms (SNP) [8]. Individually these variants have only a modest impact on CRC, but the combined effect of multiple SNPs significantly larger effects on CRC development [9, 10]. Genome-Wide Association Studies (GWAS) have led to the identification of several alleles associated with increased risk of CRC [11, 12]. Among MMR genes, MSH6 has been proposed by Wu to play a role in development of sporadic CRC [13]. Moreover, it was stated that MSH6 mutations are prone to be associated with a MSI-L phenotype than a MSI-H phenotype, which is more typical to sporadic CRC [14]. On the other hand, PMS2 gene and its polymorphism IVS1-1121C > T (rs63750451) are strongly associated with MSI-H cancers. Selective loss of PMS2 is a rare phenotype, occurring in about of 5% of all MSI-H CRCs, but this may vary according to the geographical distribution [15, 16]. Gill suggested that inactivation of *PMS2* may play an important, but limited, role in the development of sporadic MSI-H colorectal cancers [17]. In recent years, a two-antibody panel approach using PMS2 and MSH6 was proposed as an effective screening protocol for colorectal carcinoma with mismatch repair protein deficiency [18]. Authors emphasized its sensitivity in detecting MMR deficient cases, including those in which complete loss of MMR system was not present. These reports prompted us to investigate if common polymorphisms of MSH6 and PMS2 genes of MMR system modulate the susceptibility to sporadic colorectal cancer development in population of our region, including potential combine effect of these polymorphisms on risk of CRC development.

## Methods

Test DNA was isolated from peripheral blood samples collected from 200 unrelated patients with pathologically confirmed resectable CRC. Patients who meet Amsterdam criteria were excluded from the study. Control group consisted of 200 patients hospitalized in the same surgical ward for benign disease. All patients in control group has been age and sex matched and had no medical history of cancer, inflammatory bowel diseases and diabetes.

### Detection of MSH6 Gene Mutation

DNA for genotyping was isolated from blood samples of CRC patients by used a commercial kit QIAamp DNA Blood Mini Kit for isolation of high-molecular-weight DNA (Qiagen). Detection of Gly39Glu (c.116G > A) polymorphisms of theMSH6 gene was carried out by RFLP-PCR analysis performed in MultiGene TC9600-G termocycler (Labnet International, Inc. Edison, NJ, USA). Primers used for amplifications of the analysed region were as follows - forward5′-GCG CTG AGT GAT GCC AAC AAG-3′ and reverse 5′-CAG CAG GCG CTA CCG ATC TC-3′. The final products were visualized by ethidium bromide staining.

### Detection of PMS2 Gene Mutation

Blood samples genotyped for IVS1-1121C > T PMS2 polymorphisms was performed in separate PCR reactions. Samples were genotyped using the Taqman 7900HT Sequence Detection, using 10 ng genomic DNA in a 5 ml reaction using Taqman Universal PCR Master Mix (Applied Biosystems, Warrington, UK), forward and reverse primers, and FAM- and VIC-labelled probes designed by Applied Biosystems (ABI Assay-by-Design). PCR was performed in an ordinary cycler (Mx3005P, Agilent Technologies, Santa Clara, USA). After PCR, fluorescence of VIC and FAM was measured in each well using a 96-well plate-reader.

### Statistical Analysis

The statistical analysis was conducted using STATISTICA software, version 13 PL (Stat-Soft Inc.). The resulting number of each genotype was compared with the expected value based on Hardy-Weinberg equilibrium. The significance of differences between the frequencies of alleles and genotypes between groups was assessed using χ^2^ test and Fisher exact test and Cochran-Armitage test for trend. Odds ratio (OR) with corresponding confidence interval 95% (CI 95%) were calculated during multivariate regression analysis.

## Results

The study group comprised of 101 females and 99 males aged 38–90 years (median 66 years) with confirmed resectable CRC. In control group 104 females and 96 males were present, aged 35–95 years (median 64 years). Among 200 CRC patients enrolled into the study, results of genotyping were available in 192 patients for 116G > A MSH6 and 189 for IVS1-1121C > T PMS2. In control group 196 patients were successfully genotyped for MSH6 and 192 for PMS2 polymorphisms.

Distributions of genotypes and alleles in the CRC and control group for 116G > A and IVS1-1121C > T are presented in Tables 1 and 2 respectively. G allele for the MSH6 116G > A and C allele for PMS2 IVS1-1121C > T have been used in our study as references in calculations.

**Table 1:** Genotypes and alleles distribution of Gly39Glu (c.116G > A) polymorphism of*MSH6 gene* in patients with colorectal cancer (CRC) and in control group

Genotype allele	CRC patients (*n* = 192) Number (Frequency)	Control group (*n* = 196) Number (Frequency)	OR (CI 95%)	*p*
Additive model				
G/G	115 (0.60)	142 (0.72)	1 Ref.	
G/A	71 (0.37)	51 (0.26)	1.69 (1.1–2.61)	0.0216
A/A	6 (0.03)	3 (0.02)	2.08 (0.52–8.42)	0.3336
G	301 (0.78)	335 (0.85)	1 Ref.	
A	83 (0.22)	57 (0.15)	1.57 (1.04–2.38)	0.007

**Table 2:** Genotypes and alleles distribution of IVS1-1121C > T polymorphism of *MSP2 gene* in colorectal cancer (CRC) patients and in control group

Genotype allele	CRC group (*n* = 189) Number (Frequency)	Control group (*n* = 192) Number (Frequency)	OR (CI 95%)	*p*
Additive model				
C/C	121 (0.64)	124 (0.65)	1 Ref.	
C/T	62 (0.33)	64 (0.33)	0.98 (0.72–2.08)	0.914
T/T	6 (0.03)	4 (0.02)	1.54 (0.64–1.50)	0.540
C	304 (0.80)	312 (0.81)	Ref.	
T	74 (0.20)	72 (0.19)	1.05 (0.74–1.51)	0.783

According to Hardy-Weinberg chi-square analysis, distribution of genotypes and alleles in CRC group and control group for 116G > A was not consistent with H-W equilibrium (χ^2^ = 1.60 *p* = 0.786 and χ^2^ = 0.43 *p* = 0.511 respectively). Similarly, distribution of genotypes and alleles in CRC group and control group for IVS1-1121C > T was also not consistent with H-W equilibrium (χ^2^ = 0.32 *p* = 0.566 and χ^2^ = 1.69 *p* = 0.193 respectively).

Risk of CRC was approximately two-fold higher for the G/A genotype (OR 1.69 95% CI: 1.1–2.61 *p* = 0.0216) and A allele (OR 1.57 95%CI: 1.04–2.38 *p* = 0.007) (Table 1). Presence of G/A genotype was significantly higher in CRC group aged >60 years than in aged matched control group (OR 2.74 95% CI: 1.27–5.93 *p* = 0.0097). Also female patients in CRC group had significantly more often carried G/A genotype (OR 2.25 95%CI: 1.22–4.14 *p* = 0.0098).

No association was observed between the genotypes and alleles of IVS1-1121C > T polymorphism and the development of CRC in analysed population (Table 2). No significant difference in alleles or genotypes distribution for this polymorphism was observed between age and sex matched CRC and control groups.

Subsequently, we investigated the mutual influence of analysed polymorphisms on risk of CRC development. The simultaneous occurrence of the G/A genotype of the MSH6 gene and the C/C genotype of the PMS2 gene was found to increase the risk of colorectal cancer (OR = 1.78 95% Cl: 1.07–2,98 *p* = 0.029). Furthermore, co-presence of A allele of MSH6 gene and C allele of PMS2 gene was found to be significantly associated with higher risk of CRC development (OR: 2.18 95% Cl: 1.45–3.28) (Table 3).

**Table 3:** The distribution of genotypes and the analysis of the odds ratio (OR) for gene-gene interactions: Glu39Gly (c.116G > A) MSH6 and and IVS1-1121C > T PMS2 in patients with colorectal cancer (CRC) and the control group

Genotype Allele		CRC group (*n* = 187) Number (Frequency)	Control group (*n* = 192) Number (Frequency)	OR (CI 95%)	*p*
hMSH6	hPMS2	Additive model			
G/G	C/C	70 (0.37)	92 (0.48)	1 Ref.	
G/G	C/T	41 (0.21)	44 (0.23)	0.82 (0.51–1.33)	0.464
G/G	T/T	3 (0.02)	2 (0.01)	1.57 (0.26–9.47)	0.680
G/A	C/C	46 (0.24)	30 (0.16)	1.78 (1.07–2.98)	0.029
G/A	C/T	18 (0.10)	19 (0.095)	0.98 (0.50–1.93)	0.547
G/A	T/T	3 (0.02)	2 (0.01)	1.05 (0.74–1.51)	0.783
A/A	C/C	3 (0.02)	2 (0.01)	1.55 (0.26–9.38)	0.681
A/A	C/T	3 (0.02)	1 (0.005)	3.11 (0.32–30.21)	0.366
A/A	T/T	0 (0)	(0)	-	-
G	C	175	185	1 Ref.	
G	T	65	67	0.93 (0.64–1.37)	0.770
A	C	70	52	2.18 (1.45–3.28)	0.0001
A	T	24	22	1.07 (0.59–1.95)	0.879

## Discussion

Colorectal cancer is one of the most common cancers worldwide. Over the past few years, there is more and more evidence that CRC is a very heterogeneous disease and that numerous molecular and genetic features may contribute to its development and prognosis, which is especially evident in sporadic colorectal cancer [3, 9, 19]. It was also confirmed that the CRC risk is also largely accounted for by common, low penetrance alleles, known as SNPs, which either predispose directly to colorectal tumourgenesis or may have an additive effect on development risk [20, 21]. We investigated role of two such SNPs in genes responsible for MMR system. This is so far the largest study analysing the MMR genes polymorphisms in polish population. Results indicate that polymorphisms in investigated genes may influence the MSI status. Heterozygosity for 116G > A MSH6 were significantly more prevalent in CRC group of patients above 60 years old and of female gender. Those features are typical for tumours presenting the phenotype of high MSI. In case of IVS1-1121C > T polymorphism individual influence of CRC risk was not observed, but homozygosity for wild-type allele has mutual effect on increasing CRC risk in analysed group. Moreover, heterozygosity for A allele in MSH6 and homozygosity for C (wild type) allele in PMS2 were significantly more prevalent in proximal colon (unpublished data) in this population. Interpretation of the consequences of mutations in MMR genes is a difficult matter. Since connection between polymorphic variants of MSH6 and PMS2 to the MSI status is not fully established yet, it is to be answered how much mutations in these genes corresponds to deficiency in MMR system. The consequences of missense and silent mutations even in “hot” genes regions are not so obvious. Mutations in MSH6 result in a partial loss of mismatch repair and function of MSH6 gene can be overlap by MSH3 gene [22, 23]. In this regard, MSI status would have been low or stable and did not result in clinical phenotype. Studies of Brendt observed positive association between the variant and rectal cancer, where MSI-H is rare [24]. MSI-H sporadic colorectal tumours in general have usually lost MLH1 expression resulting in complete MMR deficiency. Neither MSH6, nor PMS2 polymorphisms would be though significant in case of MLH1 gene inactivation. On the other hand, one should be aware that in analysed population might be patients with somewhat atypical CRC kindreds with delayed onset of the cancer, which obscured the familial background. Moreover, familial medical history for CRC and hereditary cancers were not always transparent in dominating age group. However, an association between MSI-H CRCs and female sex, noted in our study, is valid only when MSI-H CRCs are sporadic. Nevertheless, it seems plausible that *MSH6* 116G > A polymorphism could explain at least part of the sporadic cases without family history and clearly pathogenic mutations. Because MSI status was not determined, we cannot be exactly sure that all of increased risk associated with G/A are purely correlating to MSI status. Although some features typical for MSI-H were observed in G/A genotype, the remaining ones related to clinical and pathological presentation were not investigated in this part of the study. It is hard than to assess whether these cancers were truly MSI-H. That fair amount of sporadic cancers may still demonstrate some levels of MSI having other genetic changes that correspond more to the final presentation like CIN, CIMP and BRAF status [3, 7]. The inheritance of variations in MMR genes may influence individual susceptibility to the development of colorectal cancer [21, 24], but we cannot exclude the possibility that other alterations might be present. Campbell et al. observed effect modification between MMR variants and lifestyle factors that contribute to higher CRC risk overall [25]. Similarly, Berndt and al reported that processed meat intake modified risk of colon cancer from two common variants in another MSH3 gene of MMR system [24]. It is though evident that additional studies are needed to confirm these observations and to evaluate the functional effects of these MSH6 and PMS2 variants on mismatch repair capacity.
